# The Effectiveness of Laser-Assisted Surgical Excision of Leukoplakias and Hyperkeratosis of Oral Mucosa: A Case Series in A Group of Patients

**DOI:** 10.3390/ijerph16020210

**Published:** 2019-01-13

**Authors:** Dorina Lauritano, Alberta Lucchese, Federica Gabrione, Dario Di Stasio, Javier Silvestre Rangil, Francesco Carinci

**Affiliations:** 1 Department of Medicine and Surgery, Centre of Neuroscience of Milan, University of Milano-Bicocca, 20126 Milan, Italy; f.gabrione@campus.unimib.it; 2 Multidisciplinary Department of Medical and Dental Specialties, University of Campania- Luigi Vanvitelli, 80138 Naples, Italy; alberta.lucchese@unicampania.it (A.L.); dario.distasio@unicampania.it (D.D.S.); 3 Department of Stomatology, University of Valencia, University Hospital Dr Peset, 46017 Valencia, Spain; javier.silvestre@uv.es; 4 Department of Morphology, Surgery and Experimental Medicine, University of Ferrara, 44121 Ferrara, Italy; crc@unife.it

**Keywords:** oral surgery, oral medicine, leucoplakia, hyperkeratosis, laser

## Abstract

*Introduction*: In the different branches of dentistry, the use of laser to solve different clinical situations is increasing due to numerous advantages that have been studied in literature since the 70s. Leucoplakia and hyperkeratosis can benefit from laser-assisted treatment. In most cases biopsy sampling, histological examination and, if no malignant cells are present, the follow-up is needed. However, even if the lesion is free of dysplasia patients often ask to eliminate these white spots that are always a cause of concern. *Aim*: From these numerous requests comes the idea of setting up a laser-assisted protocol as less invasive as possible to be offered to patients. The aim of the study is to find a laser-assisted protocol for the surgical excision of leucoplakia and hyperkeratosis that can both improve the clinical aspect of the lesion and be sustainable for patients. The null hypothesis has been identified in the following statement: the treatment is effective and efficient at the same time; where effectiveness was tested with the following criteria: size of the lesion, tactile perception, discomfort, pain; and efficiency with the following criteria: pain and discomfort perceived during the treatment. *Materials and methods*: To collect all data, a specially designed medical record was used. The diode laser was used with a pulsed mode and the maximum power corresponds to 1.8 W. No anaesthesia was used. Before laser-assisted treatment, the fibre was activated and was used with a contact overflowing. *Results*: Our results show a decrease in the size of the lesion statistically significant. No pain was referred during treatment, except for a slight burning sensation. *Conclusion*: In conclusion we can state that the treatment is both efficient and effective.

## 1. Introduction

According to the World Health Organization, leucoplakia is defined as a “white patch that cannot be associated either clinically or histopathologically to other diseases” so the diagnosis is by exclusion.

Leucoplakia is the most common oral precancerous alteration and appears as a white, chronic lesion, that cannot be removed with rubbing. Leucoplakia is characterized by an abnormal keratinization of the mucosa. The prevalence in the general population ranges from 0.6% to 5% and is more frequent in those between 40 and 50 years old [1]. The cause is multifactorial and there are several predisposing factors such as: mechanical irritation, dental materials causing galvanic currents, contact with carcinogens such as tobacco and alcohol and gastroesophageal reflux [2,3]. The predisposition to malignant transformation seems associated to a higher CD8+ cells levels in premalignant lesion [4]. 

The presumed diagnosis is based on the anamnesis and the clinical aspect, while the diagnosis can only be confirmed after both an incisional and excisional biopsy and its subsequent histological examination [5]. 

Some epidemiological studies have identified the occasional possibility of spontaneous regression of leucoplakias. However, in the vast majority of cases various surgical and non-surgical treatments are proposed such as elimination of the chronic irritative factors, surgical treatment with a scalpel, electrocautery, laser or cryosurgery; conservative treatment is to be considered only if the patient denies consent to surgery or if the areas have a low probability of malignant transformation [6,7,8,9,10].

Hyperkeratoses are benign lesions that usually appear in areas subjected to frictional trauma, for example in adherent gingiva under the prosthetic flanges, the retromolar trigon and the edge of the tongue, where the patient often tends to bite. During intraoral examination, hyperkeratosis tends to have a verruciform or corrugated appearance. Histological examination shows hyperkeratosis, acanthosis, hypergranulosis and inflammation of the stroma.

Although it is benign, some precancerous lesions such as leucoplakia can mimic the characteristics of the hyperkeratosis. The clinical appearance of the lesion can help clinicians in the differential diagnosis between leucoplakia and hyperkeratosis; in fact hyperkeratosis shows less definite margins than the first. Investigating the habits of the patient could be helpful [11,12]. Follow-ups must be on-going even if the histology does not manifest dysplasia, since hyperkeratosis could evolve towards a malignant transformation due to the reoccurrence of the trauma.

It has been many years since laser therapy has been introduced in dentistry and in particular in periodontal field where it succeeded in eliminating pathogenic bacterial niches in inaccessible areas, such as deep pockets, root concavities and furcation areas in a less- traumatic way [13]. One of the most effective laser therapy in decontaminating periodontal pockets and in giving improvement in CAL, PPD and BOP was the photodynamic therapy based on the use of three components: light, oxygen free radicals and photosensitizer [14,15,16,17]. 

Another option in the treatment of chronic periodontitis is the use of a desiccant agent as an adjunct to scaling and root planing (SRP). This protocol resulted in a greater reduction in clinical, microbial and inflammatory mediators compared to SRP alone [18]. 

The aim of the study is to find a laser-assisted protocol for the surgical excision of leucoplakia and hyperkeratosis that can both improve the clinical aspect of the lesion and be sustainable for patients. The null hypothesis has been identified in the following statement: the treatment is effective and efficient at the same time; where effectiveness was tested with the following criteria: size of the lesion, tactile perception, discomfort and pain; and efficiency with the following criteria: pain and discomfort perceived during the treatment.

## 2. Materials and Methods

The study group consists of 20 patients affected by leucoplakia and hyperkeratosis selected between October 2017 and March 2018 at the Dr. Peset University Hospital in Valencia and which have been subjected to a laser treatment.

The sample was selected following specific inclusion and exclusion criteria.

### 2.1. Inclusion Criteria

Execution of a biopsy and following histopathological examination in order to have the certainty to select only leucoplakias and hyperkeratosis,

Possibility of completing the clinical trial.

### 2.2. Exclusion Criteria

Histopathological presence of dysplasia,

Systemic diseases such as HIV, HBV and HCV infections, celiac disease or physiological conditions such as pregnancy,

Having performed treatment for hyperkeratosis and leucoplakia in the 2 months prior to the clinical trial.

During the first visit clinical data of each patient was collected and the informed consent to the treatment was signed. The patients were also asked about their habits, in order to identify those, which could be detrimental. The study was approved by the Clinical Research Ethics Committee (CEIC) (code 09/093), following the principles of Helsinki for human experimentation.

After collecting this data and having examined the patient both from a systemic and dental point of view, an examination of the lesions was carried out, thus evaluating:

- The site presenting the lesion,
- The histological examination,
- The size of the lesion, in order to have this data, a periodontal probe was used and the greatest distance between the sides of the lesion was measured,
- How long the lesion was present.

As previously stated, the biopsy and its subsequent histological examination were fundamental: the presence of cellular atypia, dysplasia or malignant lesions could be excluded, thus making the patient suitable for receiving laser treatment.

The biopsy was performed at the most significant point of the lesion, in order to also remove a part of the healthy tissue, useful for identification in the subsequent histological examination. First of all a 1.8 mL vial of mepivacaine and adrenaline 1:100,000 (OPTOCAIN, Molteni Dental, Milan, Italy) at the perilesional level were inoculated. After having disinfected the oral mucosa with 0.2% chlorhexidine, the part of tissue to be extracted was delimited with a 4 or 5 mm biopsy punch and subsequently extracted with a scalpel, taking care to remove all the layers of the mucosa. A suture was carried out with simple detached stitches in 4/0 silk, which was removed 7 days later.

The sample was fixed in 10% formalin (BIO-OPTICA, Milan, Italy) and sent to the pathological anatomy laboratory of the Dr. Peset University Hospital in Valencia. Here it was included in paraffin then 5-micron thick sections were made, stained with haematoxylin and eosin and analysed under an optical microscope. Approximately two weeks later the histological examination report was received.

The treatment consisted of four sessions a week and a follow-up visit one month after the last treatment session. *Surgical technique*: the surgical technique provided no anaesthesia and a superficial and fast touch of the lesion, in this way we could observe the removal of the pathological area from the underlying mucosa, which was then removed manually with the aid of anatomical tweezers. Once the white area was removed the underlying mucosa appeared to be clinically normal and healed for second intention.

The protocol stated that at each session before treatment and in the final follow-up session:

- The lesion was measured with periodontal probe and the results were reported in mm,
- The appearance of the lesion was reported,
- The discomfort was measured with an arbitrary numerical scale from 0 to 5 and the type of discomfort that the patient perceived was noted,
- The pain caused by the lesion was measured, with an NRS scale (Numerical Rating Scale) [19],
- The tactile perception was evaluated, that is the sensation of roughness that the patient felt passing the tongue on the area where the lesion was present, with an arbitrary numerical scale from 0 to 5.

During each session some aspects that were presented during the seven days since the previous session were assessed in particular:

- Discomfort measured with an arbitrary numerical scale from 0 to 5.
- Pain caused by the lesion, measured with the NRS scale [19].
- Tactile perception, measured with an arbitrary numerical scale from 0 to 5.

To perform the treatment a diode laser was used which had a wavelength of 940 nm, the fibre had a length of 9 mm and diameter of 300 microns; a pulsed mode was selected with a Ton corresponding to Toff and corresponding to 10^−3^ s, the period corresponds to the summary of Ton and Toff that is 2 × 10^−3^ s. It is also known that the frequency and the period of every electromagnetic wave are inversely proportional to each other for the equation ν = $\frac{1}{T}$; reporting the equation in our case shows that

(1) $$v=\frac{1}{2\cdot 10^{-3}}=\frac{10^{3}}{2}=5000\;\mathrm{Hz}.$$

When using the pulsed mode, there is both a peak power and average power where the latter is defined as the percentage of Ton in the period. In this case it stands at 50%. Having set the peak power at 1.8 W, the average constant power during the emission corresponds to 0.9 W; this allows intervention without using anaesthetic devices, unless specifically requested by the patient.

Before laser-assisted treatment the fibre was activated, using the special supplied activator. Both the operator and the patient wore protective glasses. The laser was used with a contact on the mucosa for about 1 min, the tissue was then left to cool for 30 s and the whole sequence was repeated in order to perform a total of four sessions in one visit. After each visit the patient was instructed to avoid too hot and too spicy foods for a few days.

### 2.3. Statistical Analysis

The data collected during the study were inserted into a database in Excel and the statistical analysis was performed with one of its applications (Stat). The data were standardized. A test unit was used in order to find the best criteria that could best evaluate the laser- treatment. For the analyses there was an intra-examiner agreement. In the first part we analysed the characteristics of the sample, while in the second phase we focused on the data during the treatment in order to obtain information regarding both the efficiency and the sustainability of the treatment.

## 3. Results

### 3.1. Sample Characteristics

20 patients were included in this study, of which 9 were affected by hyperkeratosis and 11 were affected by leucoplakia.

**Figure 1:**
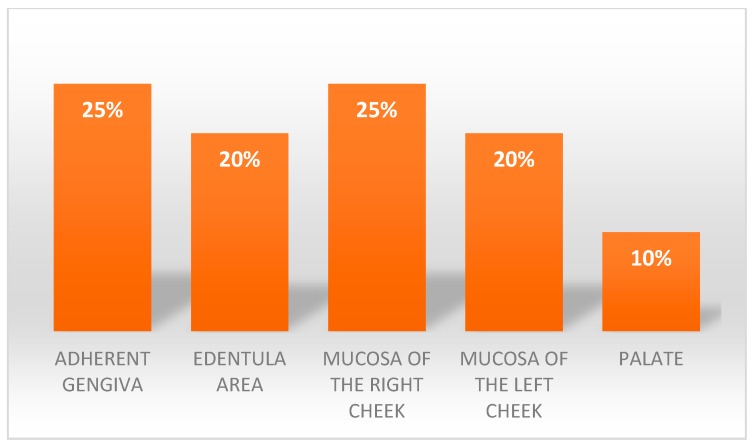
Areas of lesions’ presentation.

**Figure 2:**
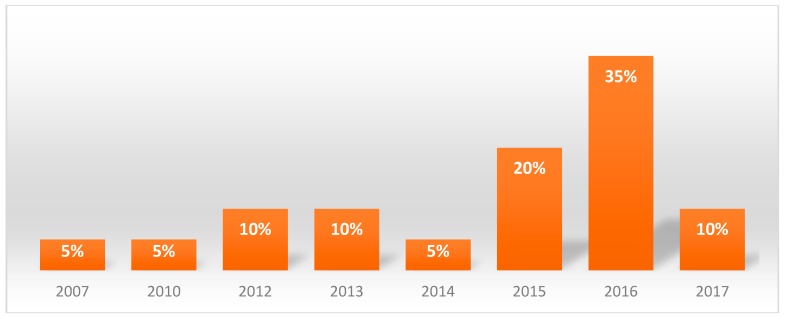
Year of onset of the lesions.

The sample consisted of 11 men (55%) with an average age of 55 years old and 9 women (45%) with an average age of 67 years old, the average age of the sample was of 61 years old. The site of onset of lesions in the oral cavity of the different patients was homogenous (Figure 1); the year of onset can be seen in Figure 2.

60% of patients had metals of dental origin such as gold; titanium or mercury derived from amalgam, 65% were carriers of both crowns and mobile prostheses and 45% of individuals had resin restorations on various elements. 35% of patients used alcoholic mouthwash, 25% had malocclusions in particular mono and bilateral cross-bite or third class and that 35% show detrimental habits, in particular episodes of morsicatio.

### 3.2. Analysis of the Lesion Size

The variation of this parameter is evaluated consecutively with one-week intervals between one observation and the next. As the dimension at time T0 was considered as an independent variable and as the variable dependent was dimension at time T1, the coefficient of the regression of this analysis was 0.81. This meant that the lesion dimension between the two time intervals taken into consideration, reduced by an average of 19%. This same reasoning was applied to the independent and dependent variables respectively before T1 and T2, then T2 and T3 and finally T3 and T4. The coefficients of the regression analysis obtained are, in order, 0.39; 0.60; 0.80. The size between the different time intervals is reduced on average by 61%, 40% and 20% respectively. The P value was always inferior to 0.05% (Table 1).

### 3.3. Discomfort Analysis

**Figure 3:**
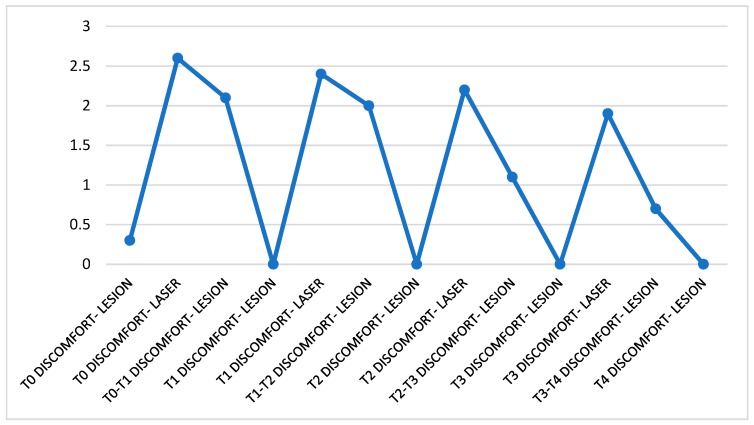
Trend of discomfort perceived throughout the treatment. The data were divided as follow: discomfort felt before the patient was subjected to treatment, during irradiation of the lesion and in the days following the session. The data refer to the entire month of observation.

The perception of the discomfort caused by the lesion before the laser therapy results, on average, in the scale selected for the measurement of this parameter, approximate to zero. The discomfort perceived during the treatment stands at average values (between 2.5 and 2.0) and tends to decrease less evidently in the short term, while in the medium to long term it follows a decreasing trend from values of 2.6 at T0 to 1.9 at T4. The values show that on average the variable assumes significant values for all observations and that the trend decreased particularly between the second and third week of treatment. The discomfort was always described as a burning sensation (Figure 3).

### 3.4. Tactile Perception

**Figure 4:**
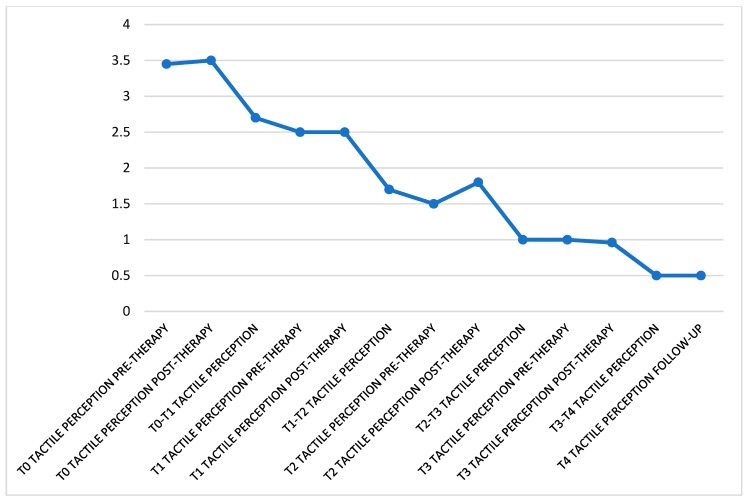
In this graph can be seen the trend of tactile perception perceived throughout the treatment. The data were divided as follow: perception felt before the patient was subjected to treatment, right after the irradiation of the lesion and in the days following the session. The data refer to the entire month of observation.

It remains substantially unchanged between before and during the treatment but the overall tendency of the values is to decrease almost constantly over time, starting at an average of 3.5 at T0 to 0.5 at T4 (Figure 4).

### 3.5. Pain: Not Perceived

As previously stated in this study both patients with simple hyperkeratotic lesions and patients with leucoplakia were included. In order to highlight if the treatment has a different efficacy between the two lesions, the patients were divided into two groups taking into consideration the nature of the lesions. For each group we analysed the same parameters used in the previous paragraphs and then we compared the results. Regarding discomfort, tactile perception and pain there are not substantial differences neither between the two groups nor between the groups and the general trend previously described. The only difference is in the variation of the size: leucoplakia follows the general trend, in fact the size reduced by 17% between T0 and T1, between T1 and T2 the size reduced by 63%, between T2 and T3 a variation of 39% occurred and finally between T3 and T4 there was a variation of 36%; in all cases the results were statistically significant. With regard to patients with hyperkeratosis, the dimensional variation shows a constant reduction during all the observations, the data showed: between T0 and T1 there was a reduction of 20%, between T1 and T2 the dimension varied by 28% and continued to do so in the following weeks of observation.

## 4. Discussion

Laser-assisted treatment has some advantages compared to traditional surgical techniques.

The main advantage common to all types of laser treatment is photocoagulation of lymphatic, hematic and nerve endings thus giving less intraoperative bleeding, less oedema and post-intervention pain. The placement of sutures is rarely necessary.

The diode laser is not indicated as the main laser for soft tissue surgery but its versatility of use led us to choose it for the study.

The diode laser is a semiconductor laser and exists in different wavelengths: from 980 nm that has greater cutting capacity and fibres that do not need to be activated, up to 810 nm which has a more biostimulatory capacity. This laser therefore has dual functionality [14,17,20]. The protocol used in this study aimed to exploit both the cutting capacity and the biostimulatory capacity; in particular, the latter was always used after the surgical phase. This helped to further reduce postoperative discomfort, oedema and to have a better, faster healing without retractable cicatrized outcomes or functional outcomes. Studies report that the laser beam activity on myofibroblasts result in reduced proliferation which results in minor dysfunctional outcomes and furthermore impacts on vessel proliferation, the synthesis of collagen and the anti-inflammatory capacity. These effects are closely related to the laser settings (fibre, power, exposure time). The best results, in terms of healing, occur with a fluency of about 4 J/cm^2^ [21,22,23,24]. 

Some studies refer to the use of lasers to treat leukoplasic and hyperkeratotic lesions but the various studies do not report the same parameters with regard to power settings, exposure time and application distance and fibre diameter [9,10,25,26,27]. 

Consequently, in our study it was difficult to compare our parameters with previously reported in the literature, because of a non-homogeneous number of terms of comparison.

Other studies reported the evaluation of the recurrence rate and the rate of malignant transformation [1,7,23,28], however no mention about the efficacy of the treatment in the short term, sustainability or invasiveness was reported.

Our protocol involves the use of the diode laser in pulsed mode with a Ton and a Toff of 110^−3^ s and a frequency of 5000 Hz. These settings were chosen in order to allow, during the emission phase of the ray, a thermal relaxation time corresponding to the Toff, which resulted in diminished overheating of tissues.

The pulsed mode was chosen in order to avoid the harmful overheating of the tissue already mentioned above; this mode allowed us to alternate a lower average power, that corresponded to the percentage of Ton in the period, with a high power peak equal to the maximum power set. In our protocol the percentage of Ton in the period is 50%, thus setting an average power of 0.9 W and a peak power of 1.8 W. With these settings anaesthesia was not required to perform the treatment unlike all the other protocols found in literature.

In order to prevent the treatment from becoming unsustainable for the individual, it was decided to irradiate the tissue for a relatively short time (about 1 min) and then leave 30 s of relaxation. This was a variation of what was found in the literature where the irradiation time was on average over 8 min [8,9,23,29].

To evaluate the effectiveness of laser-assisted treatment, the following parameters and their behaviour were taken into account during the observation period:

○ Size of the lesion.
○ Tactile perception.
○ Discomfort.
○ Pain.

The treatment was defined effective if there was a decreasing trend of all the parameters listed above, partially effective if only some parameters reduced or not effective if, compared to the beginning of the observation, the parameters have remained unchanged.

In order to evaluate the efficiency of the treatment and its sustainability for the patient, the pain and discomfort perceived during the irradiation of the lesion were considered. Treatment is considered sustainable for the patient if these parameters show medium-low values and have a decreasing trend during the four weeks of observation, if this does not happen the treatment is considered unsustainable for the patient.

### 4.1. Effectiveness

Analysis of the data shows that treatment can be defined as effective in 15 patients (75%), partially effective in 5 subjects (25%) and not effective in any of the individuals.

In five patients, belonging to the group where the treatment can be defined as effective, the lesion had completely disappeared in four weeks; while in two individuals the lesion had regressed already during the third week.

In those where the treatment is partially effective, the size of the lesion and the discomfort remained constant. In these cases the lesion did not decrease in size in a clear way but the whitish pigmentation appeared less intense.

### 4.2. Efficiency

The treatment can therefore be considered efficient for the following reasons: first, the values reported by the subjects are average values and their trend is, even if slightly, decreasing; all patients completed the clinical trial and reported less post-intervention discomfort respect to the initial biopsy performed with a blade.

Because no anaesthesia was administered and so neither adrenalin, there were the possibility of having an intraoperative bleeding that could compromise the visibility of the operative field. Thanks to the use of the laser we could provide a bloodless operating field with an excellent visibility of the lesion margins and a smoother treatment, defined the low invasiveness of laser-assisted treatment.

Since the margins were well photocoagulated, no stitches were placed but the area was left to heal by second intention.

After seven days, the complete epithelialization of the area was observed in almost all cases, only in two cases there was still presence of fibrin deposits. None of the patients had an infection of bacterial origin of the wound.

At the end of the treatment there were no retrospective cicatricle outcomes. Regard to lesions that were placed in areas of functional interest, such as the labial commissure, they did not report any functional existences of any kind.

**Figure 5:**
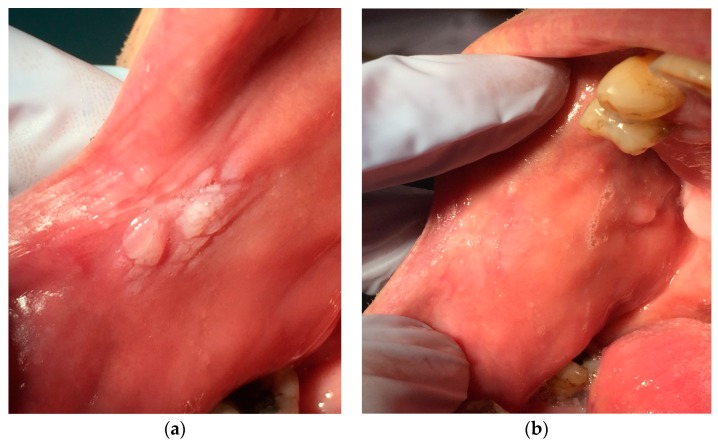
(**a**) Hyperkeratosis during the first visit: the area appears whitish and irregular, (**b**) The same area four weeks after treatment.

**Figure 6:**
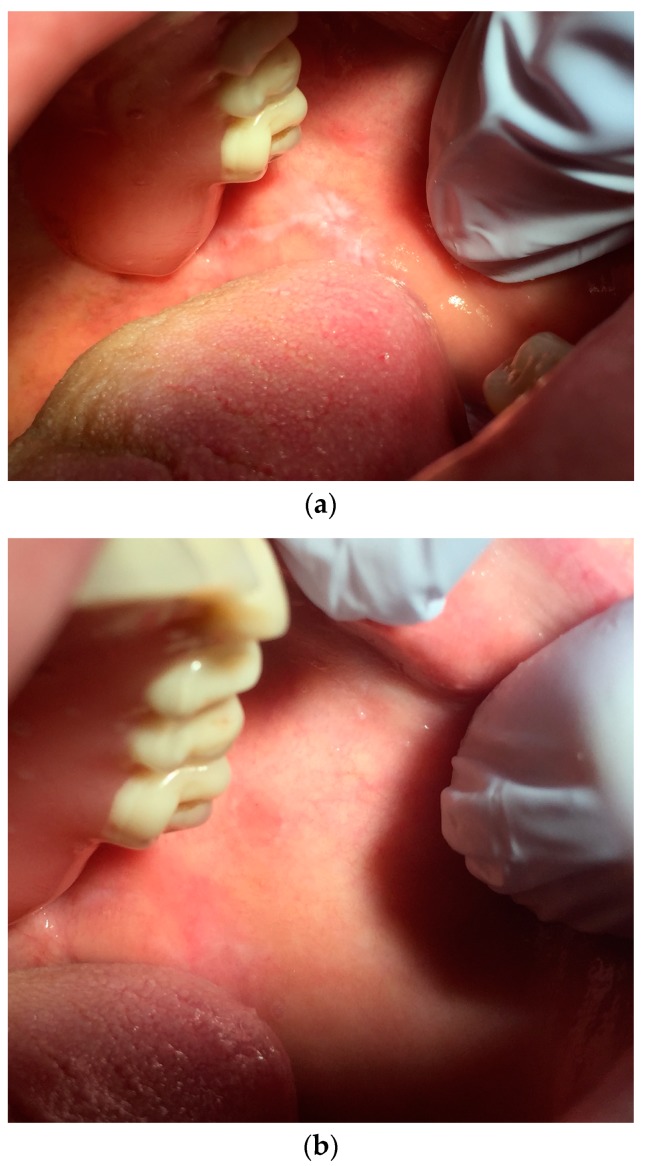
(**a**): Mucosa of the left cheek of a patient affected by leucoplakia right after the removal of stitches positioned after biopsy sampling. (**b**): Same area three weeks after laser treatment.

**Figure 7:**
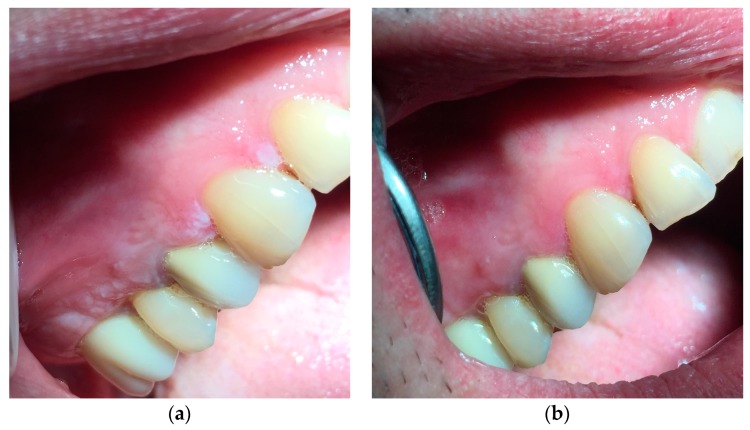
(**a**) Whitish and irregular area in the gingival mucosa. (**b**) Three weeks after the beginning of the treatment.

In the following figures the data from lesions treated with our protocol at time T0 and four weeks later, at time T4 (Figure 5a,b, Figure 6a,b and Figure 7a,b)

## 5. Conclusions

On the basis of our results, laser-assisted treatment can be considered successful. In the future it will be possible to recruit more patients in order to correlate systemic factors, such as diabetes, anaemia or gastroesophageal reflux, with the effects of the treatment in order to evaluate if those factors influence the result of the therapy. Another aspect to consider could be the rate of recurrence and malignant transformation in a longer follow-up; in this study we follow the patients for only one month after the end of the treatment and no significant differences were found since the last irradiation.

In conclusion we can state that the treatment is a valid alternative to conventional surgery with a scalpel if the lesion is bigger than 8 mm, below this limit it is better to completely eliminate the lesion during a biopsy. Lesions with a maximum size of 2 cm can regress during the four sessions set, obviously the larger the lesion, the more time needed to allow the lesion to regress.

## Figures and Tables

**Table 1:** Regression analysis, the parameter that has been used was: coefficient, significance and coefficient of determination.

Regression: Lesion Dimension T1		
	Constant	Lesion Dimension T0
Coefficient	−8.5327	0.814990
Std error of coef	11.7132	0.03492
t-ratio	−0.7285	23.3347
Significance	4756.97%	0.0000 %
Beta-weight		0.9839
Standard error of regression		42.4721
Coefficient of determination		96.80%
Adjusted coef of determination		96.62%
Number of observation		20
Residual degrees of freedom		18
t-statistic for computing		
95% confidence intervals		2.1009

## References

[1] Bokor-Bratić M. (2003). Prevalence of oral leucoplakia. Med. Pregl..

[2] Gönen Z., Asan C.Y., Etöz O., Alkan A. (2016). Oral leucoplakia associated with amalgam restorations. J. Oral Sci..

[3] Kazanowska-Dygdala M., Dus I., Radwan-oczko M. (2016). The presence of Helicobacter pylori in oral cavities of patients with leucoplakia and oral lichen planus. J. Appl. Oral Sci..

[4] Chaves A.L.F., Silva A.G., Maia F.M., Lopes G.F.M., de Paulo L.F.B., Muniz L.V., Dos Santos H.B., Soares J.M.A., Souza A.A., de Oliveira Barbosa L.A. (2018). Reduced CD8+ T cells infiltration can be associated with a malignant transformation in potentially malignant oral epithelial lesions. Clin. Oral Investig..

[5] Ardiuno P.G., Bagan J., El-Naggar A.K., Carrozzo M. (2013). Urban legends series: Oral Leucoplakia. Oral Dis..

[6] Kumar A., McCaul L.C.J., Kerawala C.J., Coombed D., Godden D., Brennan P.A. (2013). How should we manage oral leucoplakia?. Br. J. Oral Maxillofac. Surg..

[7] Monteiro L., Barbieri C., Martins M., Salazar F., Vescovi P., Meleti M., Pacheco J. (2017). Type of surgical treatment and recurrence of oral leucoplakia: A retrospective clinical study. Oral Med. Pathol..

[8] Modegas-Vegara A., Hueto-Madrid J., Chimenos-Küstner E., Bescos-Atin C. (2015). The treatment of oral leucoplakia with the CO_2_ laser: A retrospective study of 65 patients. J. Cranio-Maxillo-Fac. Surg..

[9] Del Corso G., Gissi D.B., Tarsitano A., Costabile E., Marchetti C., Foschini L.M. (2015). Laser evaporation versus laser excision of oral leucoplakia: A retrospective study with long-term follow-up. J. Cranio-Maxillo-Fac. Surg..

[10] Chainani-Wu N., Madden E., Sim C., Collins K., Silverman S. (2015). Clinical predictors of oral leucoplakia recurrence following CO_2_ laser vaporization. J. Cranio-Maxillo-Fac. Surg..

[11] Grammer R., Lerman M.A. (2014). Keratosis of unknown significance and leucoplakia: A preliminary study. Oral Maxillofac. Pathol..

[12] Bellato L., Martinelli P., Lombardi T. (2013). Alveolar ridge keratosis—A retrospective clinicopathological study. Head Face Med..

[13] Matarese G., Ramaglia L., Cicciù M., Cordasco G., Isola G. (2017). The Effects of Diode Laser Therapy as an Adjunct to Scaling and Root Planing in the Treatment of Aggressive Periodontitis: A 1-Year Randomized Controlled Clinical Trial. Photomed. Laser Surg..

[14] Caccianiga G., Rey G., Paiusco A., Lauritano D., Cura F., Ormianer Z., Carinci F. (2016). Oxygen high level laser therapy is efficient in treatment of chronic periodontitis: A clinical and microbiological study using PCR analysis. J. Biol. Regul. Homeost. Agents.

[15] Yadwad K., Veena H.R., Patil S., Shivaprasad B.M. (2017). Diode laser therapy in the management of chronic periodontitis—A clinico-microbiological study. Int. Med. Appl. Sci..

[16] Meimandi M., Ardakani M., Nejad A., Yousefnejad P., Saebi K., Tayeed M. (2017). The Effect of Photodynamic Therapy in the Treatment of Chronic Periodontitis: A Review of Literature. J. Lasers Med. Sci..

[17] Roncati M., Lauritano D., Cura F., Carinci F. (2016). Evaluation of light-emitting diode (LED-835 NM) application over human gingival fibroblast: An in vitro study. J. Biol. Regul. Homeost. Agents.

[18] Isola G., Matarese G., Williams R.C., Siciliano V.I., Alibrandi A., Cordasco G., Ramaglia L. (2018). The effects of a desiccant agent in the treatment of chronic periodontitis: A randomized, controlled clinical trial. Clin. Oral Investig..

[19] Sirintawat N., Sawang K., Chaiyasamut T., Wongsirichat N. (2017). Pain measurement in oral and maxillofacial surgery. J. Dent. Anesth. Pain Med..

[20] Usama R.K., Onkar S., Birangane R., Chaudhari S., Kulkarni A. (2015). Treatment of oral leucoplakia with diode laser: A pilot study on Indian subjects. Asian Pac. J. Cancer Prev..

[21] Kuribayashi Y., Tsushima F., Morita K., Matsumoto K., Sakurai J., Uesugi A., Sato K., Oda S. (2015). Long-term outcome of non-surgical treatment in patients with oral leucoplakia. J. Cranio-Maxillo-Fac. Surg..

[22] Natekar M., Raghuveer H., Rayapati D., Shobha E., Prashanth N., Rangan V. (2017). A comparative evaluation: Oral leukoplakia surgical management using diode laser, CO_2_ laser and cryosurgery. Oral Surg..

[23] Brouns E.R.E.A., Baart J.A., van der Waal I., Karagozoglu K.H., Aartman I.H.A. (2013). Treatment results of CO_2_ laser vaporisation in a cohort of 35 patients with oral leukoplakia. Oral Dis..

[24] Maloth K., Velpula N., Kodangal S., Sangmesh M., Meka S.U. (2016). Photodynamic Therapy—A Non-invasive Treatment Modality for Precancerous Lesions. J. Lasers Med. Sci..

[25] Van der Hem P.S., van der Wal J.E., Nauta J.M., Roodenburg J.L.N. (2005). The results of CO_2_ laser surgery in patients with oral leukoplakia: A 25 year follow up. Oral Oncol..

[26] Vivek V., Balan A., Jayasree R.S., Sreelatha K.T., Gupta A.K. (2008). Three-year follow-up of oral leukoplakia after neodymium: Yttrium aluminum garnet (Nd:YAG) laser surgery. Laser Med. Sci..

[27] Comacho-Alonso L.J. (2013). Comparison of pain and swelling after removal of oral leukoplakia with CO_2_ laser and cold knife: A randomized clinical trial. Med. Oral.

[28] Tambuwala A., Sangle A., Khan A., Sayed A. (2014). Excision of Oral Leukoplakia by CO_2_ Lasers Versus Traditional Scalpel: A Comparative Study. J. Maxillofac. Oral Surg..

[29] Romeo U., Russo N., Palaia G., Tenore G., Del Vecchio A. (2014). Oral proliferative verrucous leukoplakia treated with the photodynamic therapy: A case report. Ann. Stomatol..

